# Atomic Layer Deposition of AgX (X = Cl, Br, I) Thin Films

**DOI:** 10.1021/acsomega.5c06913

**Published:** 2025-11-14

**Authors:** Aida Heidari, Georgi Popov, Timo Hatanpää, Alexander Weiß, Mykhailo Chundak, Kenichiro Mizohata, Mikko Ritala, Marianna Kemell

**Affiliations:** † Department of Chemistry, 3835 University of Helsinki, Helsinki FI-00014, Finland; ‡ Department of Physics, 3835 University of Helsinki, Helsinki FI-00014, Finland

## Abstract

Silver halides (AgX, X = Cl, Br, I) are promising materials for various applications, including optical, medical, and electronic technologies, due to their light sensitivity, high refractive index, and antiseptic properties. Silver halides are also important constituents of Pb-free photovoltaic materials such as rudorffites and double halide perovskites. This study presents atomic layer deposition (ALD) processes for the deposition of silver halide thin films using (2,2-dimethyl-6,6,7,7,8,8,8-heptafluorooctane-3,5-dionato)­silver­(I)-triethylphosphine (Ag­(fod)­(PEt_3_)) as the silver precursor and different halide precursors, including TiX_4_, SnX_4_, GaX_3_, and HX (X = Cl, Br, I). The films were deposited on Si substrate at temperatures ranging from 105 to 195 °C. Grazing incidence X-ray diffraction (GI-XRD) revealed the formation of crystalline phases of AgI, AgBr, and AgCl. The choice of the halide precursor significantly affected the film morphology and purity. Titanium tetrahalides led to the most consistent growth-per-cycle (GPC) over the studied temperature range, resulting in films with superior crystallinity and purity. In particular, TiI_4_ was identified as the most effective halide precursor which led to AgI films with superior continuity and purity. Although GaX_3_, SnX_4_, and HX precursors showed good performance in terms of GPC, the resulting films exhibited significant sensitivity to the deposition temperature and contained impurities. This study demonstrates that ALD is a robust technique for the controlled deposition of silver halides.

## Graphical abstract

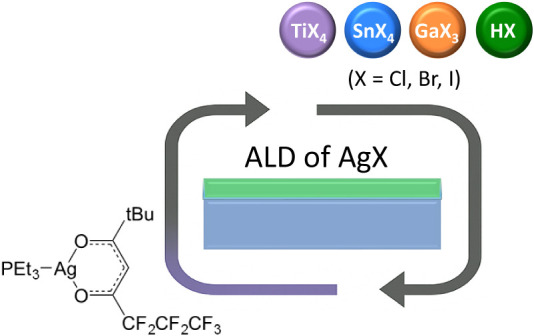

## 1 Introduction

Silver halides have attracted the attention of scientists and technologists due to their unique properties and diverse applications. Their light-sensitive nature is exploited, particularly in the field of photography, where they serve as the key component in photographic films. With refractive indices higher than 2.1, silver halide-based compounds exhibit promising characteristics for optical applications.^1–3^ Moreover, they are infrared transparent and antiseptic, which have enabled their use in niche optical, medical, and patterning applications.^2,4^ In addition to these traditional applications, silver halides are important constituents of emerging lead-free halide perovskite materials.^1,5^ Although lead-based halide perovskites have been widely studied for their remarkable optoelectronic characteristics, their widespread use has been limited by concerns regarding lead toxicity, instability in typical environmental conditions, and environmental repercussions.^5,6^ Hence, researchers have focused on identifying alternative materials by replacing lead with nontoxic cations. Silver halide containing double perovskites and silver bismuth iodide rudorffites have recently emerged as interesting candidates for different applications including solar cells,^7^ sensors,^8^ light-emitting diodes,^9^ and microelectronic components.^10,11^ To develop and optimize these materials and their devices, it is important to first deposit high-quality silver halide thin films with controlled composition and thickness.

Emulsion coating technique is one of the traditional methods used for preparing silver halide-based photographic films, wherein an emulsion containing silver halide grains is applied on a substrate material.^12^ Chemical bath deposition (CBD) and sol–gel methods are also utilized for depositing silver halide films.^13,14^ In the CBD method, silver halides are deposited through in situ hydrolysis of organic haloalcohols.^13^ In the sol–gel method, chlorine and bromine precursors and colloidal silver dispersions are applied onto a silica substrate using a spin-coating method. Subsequently, the formation of silver halide coatings occurs upon heating above 300 °C.^14^ These conventional processes encounter limitations, including poor adhesion at the film/substrate interface, as well as problems in controlling the film thickness because of the high deposition rates.^13^

Studies have focused on addressing these concerns by exploring novel synthesis techniques. Atomic layer deposition (ALD) has emerged as a promising alternative for depositing metal halides in thin film form. Its unique ability to deposit materials with atomic-scale precision, coupled with the inherent conformality and controllability of thin film growth, provides a compelling opportunity to overcome limitations of the other deposition methods.^15–17^ Moreover, ternary and quaternary compounds can be deposited by combining ALD processes of the corresponding binary compounds. In our previous studies, we employed a two-step approach to deposit CsPbI_3_ and CsSnI_3_, wherein a CsI film was initially prepared, followed by the deposition of PbI_2_ or SnI_2_ on top. In these ALD processes cesium bis­(trimethylsilyl)­amide, lead­(II) bis­[bis­(trimethylsilyl)­amide], and tin­(II) bis­[bis­(trimethylsilyl)­amide] were used as the precursors of cesium, lead, and tin, respectively, along with tin­(IV) iodide as the iodine precursor.^18,19^ Building on this strategy, the current study focuses on developing efficient ALD processes for depositing binary silver halide thin films, which is a critical foundation for extending this methodology to the fabrication of silver-based ternary and quaternary halide materials.

An important factor in ALD is the choice of precursors, which significantly affects the quality and properties of the deposited films. Several studies have reported the use of (2,2-dimethyl-6,6,7,7,8,8,8-heptafluorooctane-3,5-dionato)­silver­(I)-triethyl-phosphine (Ag­(fod)­(PEt_3_)) as an appropriate precursor coupled with hydrogen plasma as a reducing agent for the ALD of silver thin films.^20–22^ However, to the best of our knowledge, there are currently no known ALD processes for depositing silver halides. Most chemical vapor deposition (CVD) precursors of silver that possess sufficient volatility exhibit low thermal stability. On the other hand, byproducts produced from the precursors can adversely affect the ALD process. These byproducts may cause etching effects or, if nonvolatile, lead to poor crystallinity and impurities in the resulting film.^23^ To deposit high-quality and continuous silver halide films, different halide precursors should be evaluated to achieve a wide and stable ALD window that is compatible with subsequent ALD processes used for multicomponent halide materials. In this work, we developed silver halide ALD processes by employing volatile metal halides, such as TiX_4_, GaX_3_, SnX_4_, and hydrohalic acids HX (X= Cl, Br, or I), as the precursors of the corresponding halogens, and Ag­(fod)­(PEt_3_) as the silver source.

## 2 Results and Discussion

### 2.1 Comparison of Halide Precursors

#### 2.1.1 Film Deposition

We first compared the deposition of AgX films using Ag­(fod)­(PEt_3_) and various halide precursors (TiX_4_, GaX_3_, SnX_4_, and HX, X = Cl, Br, I) at deposition temperatures ranging from 105 to 195 °C. Silicon was used as a substrate for ALD of silver halides. Ag­(fod)­(PEt_3_) is a thermally stable and volatile silver precursor with a fluorinated β-diketonate (fod) and triethylphosphine (PEt_3_) ligand. According to Kariniemi et al.,^20^ its volatility and reactivity allow ALD of silver thin films within a narrow deposition temperature range (120–140 °C) where saturative growth occurs with negligible decomposition of Ag­(fod)­(PEt_3_). However, its stability may change in the gas phase and when adsorbing on different materials. Therefore, precise optimization of the deposition parameters is required.

Regarding halide precursors, TiX_4_ and SnX_4_ have been used in similar ALD processes as appropriate halogen sources. In particular, we used TiI_4_ and SnI_4_ to deposit cesium iodide films through ligand-exchange reactions.^19^ Similarly, deposition of CaF_2_ and MgF_2_ using HF^24,25^ TiF_4_,^25^ and TaF_5_ ^26^ highlights the applicability of metal halides and hydrogen halides in ALD, and their strong reactivity can be readily adapted to bromides and chlorides as well. This chemistry can also be extended to the formation of AgX films, where the halogen precursor exchanges ligands with the Ag precursor. The source temperature of Ag­(fod)­(PEt_3_) (95 °C) restricted the lowest deposition temperature. On the other hand, at deposition temperatures above 200 °C, Ag­(fod)­(PEt_3_) began to decompose, which we observed as a black residue appearing in the hot end of the precursor delivery tube.

The ALD behavior of silver halide films strongly depends on the halide precursor used, which leads to clear differences in growth per cycle (GPC). Different halide precursors can react with Ag­(fod)­(PEt_3_) in various ways, depending on their chemical nature, volatility, and Lewis acidity. While the exact reactions can vary and are not completely characterized, the ALD of silver halides generally involves the adsorption of the Ag­(fod)­(PEt_3_) precursor on the substrate surface. The adsorbed Ag­(fod)­(PEt_3_) then reacts with the halide source through ligand exchange.

For silver iodide films (Figure 1a), only TiI_4_ exhibited a relatively stable GPC (0.6–0.8 Å) over the 105–195 °C range. The GPC of silver iodide films deposited from Ag­(fod)­(PEt_3_) and iodide precursors (GaI_3_, SnI_4_, and HI) decreases with increasing deposition temperature (Figure 1a). SnI_4_ and TiI_4_ are both tetrahedral group IV metal iodides and are structurally similar. However, TiI_4_ has stronger Lewis acidity because of its higher charge density and smaller ionic radius. The superior performance of TiI_4_ can be attributed to its sufficient volatility and strong Lewis acidity that facilitate efficient ligand exchange with Ag­(fod)­(Pet_3_).

**Figure 1:**
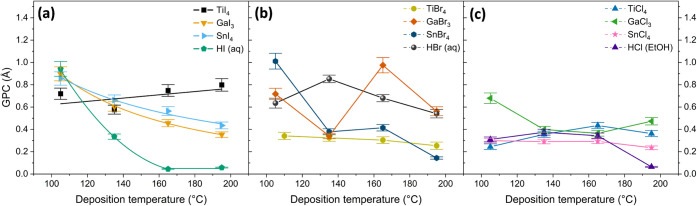
Growth per cycle of ALD (a) AgI, (b) AgBr, and (c) AgCl thin films as a function of deposition temperature using different halide precursors. Depositions were done with 800 cycles, a pulse duration of 1.5 s for both Ag­(fod)­(PEt_3_) and halide precursors, and purge durations of 1.0 s.

Silver bromide films deposited from Ag­(fod)­(PEt_3_) and SnBr_4_ precursors exhibit narrow temperature ranges with constant GPC, whereas no such ranges are observed for GaBr_3_ and HBr. SnBr_4_ shows the highest GPC (∼1.0 Å) at 105 °C, likely due to enhanced adsorption at lower temperatures. When GaBr_3_ was used as the bromide precursor, the GPC exhibited a fluctuating trend. This fluctuation indicates that the deposition process with GaBr_3_ is more complex, possibly due to competing reactions. The competing reactions lead to incorporation of gallium impurities the concentration of which can be affected by temperature. The GPC of the TiBr_4_ process was relatively constant (GPC ∼ 0.3 Å) over the studied temperature range (Figure 1b). Among transition metal bromides, TiBr_4_ possesses the highest volatility. Its characteristics lie between those of TiCl_4_ and TiI_4_, and it exhibits strong Lewis acidity.^27^ As a result, TiBr_4_ provides an effective balance of volatility, Lewis acidity, and reactivity required for self-limiting surface reactions in the ALD of silver bromide.

The GPCs of silver chloride films deposited with TiCl_4_, GaCl_3_, SnCl_4_, and HCl as halide precursors were lower than for the other halides and showed relatively limited dependence on the deposition temperature. This behavior can be partially explained by the strength of the metal–ligand bond.^28^ For most metal halides, the bond strength decreases in the order M–Cl > M–Br > M–I. For instance, Mohlala et al.^29^ have employed density functional theory (DFT) to study the thermal stability and reactivity of titanium halide-based precursors. According to their findings, thermal stability follows the order TiF_4_ > TiCl_4_ > TiI_4_, while reactivity shows the inverse order. This higher bond strength of chlorides compared to bromides and iodides supports steadier growth rates for silver chloride films. On the other hand, iodide and bromide precursors, with weaker M–L bonds, exhibit more temperature-sensitive GPC changes.

Compared to GaCl_3_ and HCl, the TiCl_4_ and SnCl_4_ precursors showed less pronounced changes in GPC as a function of deposition temperature (Figure 1c). GaCl_3_ exhibited the highest GPC at the lowest temperature. However, its GPC sharply decreased as the temperature increased to 135 °C. After a range of steady GPC between 135 °C and 165 °C, the GPC again increased with further temperature increase. Interpretation of these observations is complicated by fact that AgCl films deposited with GaCl_3_ contained high concentrations of Ga impurities. In the 135–165 °C range, the GPC values for all chloride precursors were closest to each other and changed the least.

#### 2.1.2 Film Properties

Grazing incidence XRD patterns of silver halide films deposited using the different halide precursors provide information on the crystalline properties and phase purity of the films. As shown in Figure 2a, the XRD patterns confirm the crystalline structure of the silver iodide films, with sharp peaks corresponding to the hexagonal β-AgI phase (ICDD No. 09-0374).^30,31^ Figure 2b shows the XRD patterns of the AgBr films, with diffraction peaks ascribed to the cubic AgBr (ICDD No. 06-0438).^32^ The silver bromide film deposited using GaBr_3_ shows additional reflections attributed to Ga^I,II^Br_*x*_ that are indicative of undesired redox side reactions occurring during the deposition. Apart from this, no significant differences were observed in the XRD patterns of the silver bromide films. The XRD patterns measured for AgBr films deposited using SnBr_4_ at the temperature range of constant GPC (135–165 °C) showed no notable differences (Figure S1). In contrast, there are significant differences in the XRD patterns of the silver chloride films, as shown in Figure 2c. The XRD pattern of the film made with GaCl_3_ is in a good agreement with that of randomly oriented silver chloride. The sharp diffraction peaks are indexed to the cubic AgCl (ICDD No. 31-1238).^33^ For the AgCl films deposited within the temperature range of constant GPC (135 °C and 165 °C), the (200) plane is the most prominent, showing that the crystals have a preferred orientation at these temperatures (Figure S2). XRD patterns of films deposited using TiCl_4_ and SnCl_4_ exhibit only a single (1 1 1) reflection, revealing highly oriented growth. In contrast, the film deposited with HCl displays multiple reflections related to the cubic AgCl, among which the (2 0 0) plane is the most dominant.

**Figure 2:**
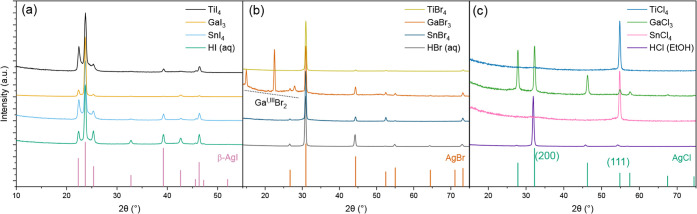
GI-XRD patterns of (a) AgI, (b) AgBr, and (c) AgCl on Si substrate. The films were deposited at 105 °C with 800 cycles. Ag­(fod)­(PEt_3_) pulse, halide pulse, and purge durations were 1.5, 1.5, and 1.0 s, respectively.

SEM imaging revealed significant differences in the continuity and morphology of the silver halide films deposited with 800 cycles. All the films had island structures still after 800 ALD cycles. Such a strong agglomeration can be explained by the low melting points of silver halides (AgCl 455 °C; AgBr 432 °C; AgI 558 °C) and consequent high mobility during the nucleation of the films. While the discontinuity may limit the use of these binary silver halide films, the ALD processes can be valuable in depositing ternary silver bismuth iodides and quaternary silver-based double-perovskites.

To quantify the film continuity, surface coverage ratios were calculated from the SEM images using ImageJ software (Table S1). Processes using TiI_4_ and SnI_4_ (surface coverage of 97% and 91%, respectively) produced the most continuous films. The films deposited using GaI_3_ and HI (aq) were discontinuous (surface coverage of 78% each) at even relatively high thicknesses (70–80 nm) (Figure 3a–d). In the case of AgBr films, achieving continuity with 800 cycles was a challenge with all the halide precursors except GaBr_3_ (Figure 3e–h). AgBr film deposited from SnBr_4_ consisted of large and irregular islands with poor surface coverage (79%). The growth behavior of this film showed a strong dependence on deposition temperature, which can be attributed to enhanced adsorption at lower temperatures (Figure 1b). Therefore, we also recorded SEM images for the films deposited at 135–165 °C (Figure S3a,b). In this temperature range, the deposited films consisted of smaller grains and had nominal thicknesses of about 30 nm. At 195 °C, the grains became even smaller, and the nominal thickness decreased to 10 nm (Figure S3c).

**Figure 3:**
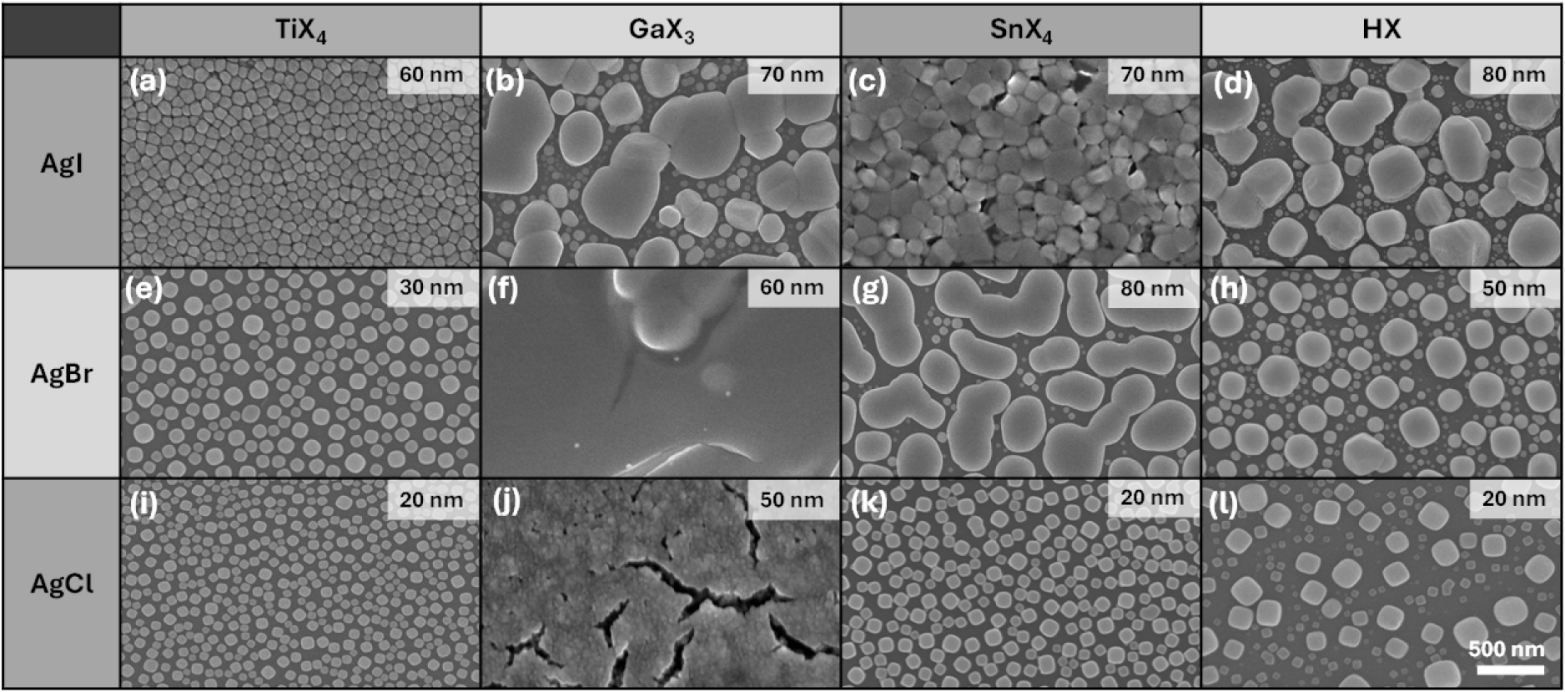
FESEM images of ALD AgI films deposited using TiI_4_ (a), GaI_3_ (b), SnI_4_ (c), and HI (d); AgBr films deposited using TiBr_4_ (e), GaBr_3_ (f), SnBr_4_ (g), and HBr (h); AgCl films deposited using TiCl_4_ (i), GaCl_3_ (j), SnCl_4_ (k), and HCl (l). All films were deposited at 105 °C on Si with 800 cycles. The Ag­(fod)­(PEt_3_) pulse, halide pulse, and purge durations were 1.5, 1.5, and 1.0 s, respectively.

Among the precursors used for AgCl film deposition, GaCl_3_ produced the thickest and continuous film at 105 °C, with a surface coverage of 98%. SEM images of AgCl films deposited from GaCl_3_ at 135, 165, and 195 °C show that the films become discontinuous at higher temperatures (Figure S4a–c) due to lower thickness. The films deposited using TiCl_4_ (Figure 3i), SnCl_4_ (Figure 3k), and HCl (Figure 3l) consist of separate particles, with surface coverages ranging from 51% to 64%. AgBr and AgCl films deposited using TiX_4_, SnX_4_, and HX are discontinuous even after 800 ALD cycles, and a critical thickness above 70–80 nm is required for full coalescence. This is consistent with a Volmer–Weber growth mode, where isolated islands should grow and merge before forming a continuous film.

Table 1 compares the characteristics of AgI, AgBr, and AgCl ALD processes based on the halide precursor choice. EDS results showed that titanium halides resulted in AgX films with no Ti impurities (Figure S5a–c). Notably, TiI_4_ produced continuous films with a thickness of 60 nm and a GPC of 0.7 Å per cycle. All the films obtained from gallium and tin halides had gallium and tin impurities, respectively. EDS detected less than 2 at.% Ga impurity in the AgI film deposited using GaI_3_ (Figure S5d), and the resulting film was discontinuous. GaBr_3_ and GaCl_3_ resulted in continuous films with relatively high GPCs (0.7 Å per cycle for both). However, EDS analysis revealed significant Ga impurities (25 at.% and 36 at.% for AgBr and AgCl films, respectively) (Figure S5e,f). As can be seen in Figure S5g,h, films deposited using SnI_4_ and SnBr_4_ exhibited much lower Sn content (<2–3 at.%). EDS measurements revealed that all processes except the ones with GaBr_3_ and GaCl_3_ produced stoichiometric silver halides.

**Table 1:** Characteristics of AgI, AgBr, and AgCl Thin Films Deposited Using Different Halide Precursors^a^

Films	Precursors	Metal residues from precursor	Continuity of films	GPC (Å/cycle)
AgI	Ag(fod)(PEt_3_)/TiI_4_	No Ti (ToF-ERDA)	Continuous at 60 nm	0.7
	Ag(fod)(PEt_3_)/GaI_3_	<2 at.% Ga (EDS)	Discontinuous at 70 nm	0.9
	Ag(fod)(PEt_3_)/SnI_4_	3 at.% Sn (EDS)	Continuous at 70 nm	0.9
	Ag(fod)(PEt_3_)/HI	-	Discontinuous at 80 nm	1.0
AgBr	Ag(fod)(PEt_3_)/TiBr_4_	No Ti (ToF-ERDA)	Discontinuous at 30 nm	0.3
	Ag(fod)(PEt_3_)/GaBr_3_	25 at.% Ga (EDS)	Continuous at 60 nm	0.7
	Ag(fod)(PEt_3_)/SnBr_4_	<2 at.% Sn (EDS)	Discontinuous at 80 nm	1.0
	Ag(fod)(PEt_3_)/HBr	-	Discontinuous at 50 nm	0.6
AgCl	Ag(fod)(PEt_3_)/TiCl_4_	No Ti (ToF-ERDA)	Discontinuous at 20 nm	0.2
	Ag(fod)(PEt_3_)/GaCl_3_	36 at.% Ga (EDS)	Continuous at 50 nm	0.7
	Ag(fod)(PEt_3_)/SnCl_4_	2 at.% Sn (EDS)	Discontinuous at 20 nm	0.3
	Ag(fod)(PEt_3_)/HCl	-	Discontinuous at 20 nm	0.3

a Deposition temperature: 105 °C; cycles: 800; Ag­(fod)­(PEt_3_) pulse duration: 1.5 s; halide pulse duration: 1.5 s; purge duration: 1.0 s.

Small amounts of oxygen were detected in most of the films, regardless of the halide precursor used. For the films deposited using hydrohalic acid solutions, oxygen incorporation may be attributed to aqueous or alcoholic solvents. However, oxygen was also present in several films deposited from the anhydrous precursors. This indicates that its incorporation most likely occurred after the deposition as a result of exposure to ambient air during sample transfer.^18^ Moreover, oxygen could originate from the metal–oxygen bonds in the Ag­(fod)­(Pet_3_).

X-ray photoelectron spectroscopy (XPS) results were in good agreement with the EDS and ToF-ERDA findings. Figures S6a–d, S7a–d, and S8a–d show the full XPS spectra of AgI, AgBr, and AgCl films deposited using TiX_4_, GaX_3_, SnX_4_, and HX precursors, respectively. All spectra revealed characteristic peaks of silver and the corresponding halogens. AgI films exhibited smaller C 1s and O 1s peaks compared to those of AgBr and AgCl. No peaks related to Ti were observed in the XPS spectra of films deposited using TiX_4_ precursors (Figures S6a, S7a and S8a). This supports the conclusion that Ti is not incorporated into the resulting films. In addition, consistent with the EDS results, no distinguishable Ga or Sn peaks were observed in the AgI films deposited from GaI_3_ (Figure S6b) and SnI_4_ (Figure S6c), due to their low concentrations. However, sharp Ga peaks were observed in the AgBr and AgCl films deposited using GaBr_3_ and GaCl_3_, which confirms the high impurity contents detected by EDS. Similarly, small Sn peaks were detected in the AgBr and AgCl films deposited using SnBr_4_ and SnCl_4_, again in agreement with the EDS results.

As XPS is a surface-sensitive technique that probes only the top ∼5–10 nm of the material, the presence or absence of substrate-related peaks can provide useful information regarding film coverage. In some films, peaks at binding energies of ∼100 eV and ∼151 eV, corresponding to Si 2s and Si 2p, were observed indicating incomplete film coverage. In the case of AgI films deposited from TiI_4_ and SnI_4_ (Figure S6a,c), and AgBr and AgCl films deposited from GaBr_3_ (Figures S7b and S8b), these Si peaks were not observed. This supports the SEM observations that the films were continuous and completely covered the silicon substrate.^34,35^

### 2.2 Ag­(fod)­(PEt_3_)—TiX_4_ Process Optimization

Titanium halides were selected as the halogen sources for further optimization based on two results. First, as illustrated in Figure 1, the GPCs of the films obtained from TiX_4_ (X = I, Br, and Cl) precursors show no dependency on the deposition temperature. Second, according to the XRD and EDS results, the films deposited with TiX_4_ precursors had no Ti impurity (Figure 2 and Table 1). Based on these advantages, this section focuses on the optimization of Ag­(fod)­(PEt_3_) -TiX_4_ processes.

Figure 4a–i presents GPCs as a function of the pulse durations of Ag­(fod)­(PEt_3_) and TiX_4_ precursors, along with film thicknesses as a function of the number of deposition cycles. In all three silver halide processes using titanium tetrahalides, the GPC saturates with respect to both precursor pulse durations. AgI requires at least a 2.0 s long Ag­(fod)­PEt_3_ pulse for saturation (Figure 4a), whereas AgCl and AgBr GPCs saturate with 1.5 s pulses (Figure 4d,g). TiI_4_, TiBr_4_, and TiCl_4_ require 2.0 s, 1.0 s, 1.5 s long pulses for saturation, respectively (Figure 4b,d,h). The saturation verifies the self-limiting nature of ALD. Figure S9 shows the effect of purge durations on GPC. For all three silver halide processes, the purge duration does not significantly affect the GPC. This shows that adequate purging is achieved at the studied purge durations.

**Figure 4:**
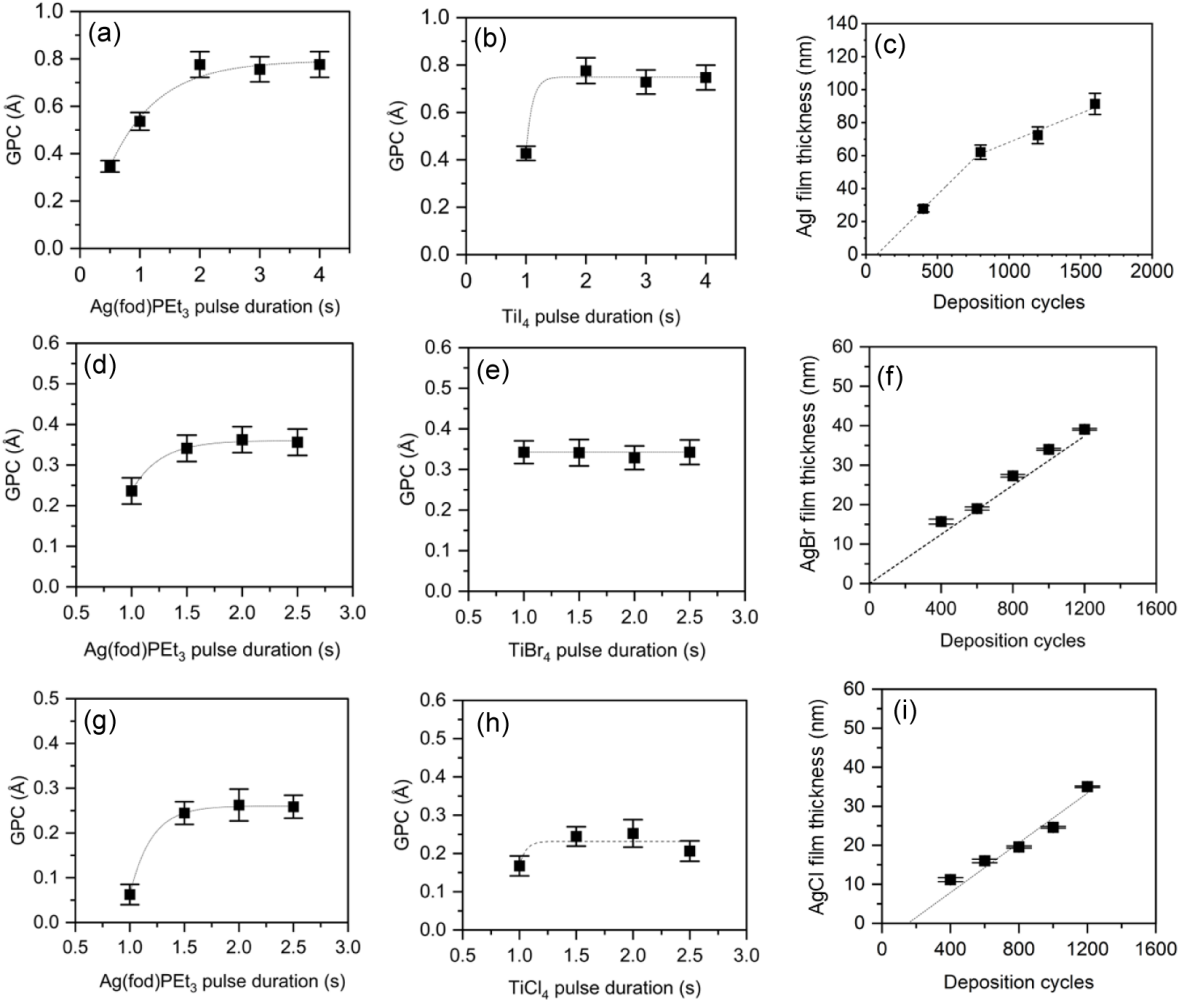
GPC of AgI thin films as a function of (a) Ag­(fod)­(PEt_3_) pulse duration and (b) TiI_4_ pulse duration. (c) AgI film thickness as a function of deposition cycles. Unless otherwise evident the depositions in (a)–(c) were made with 800 cycles of 2.0 s long pulses and purges at 105 °C. GPC of AgBr thin films as a function of (d) Ag­(fod)­(PEt_3_) pulse duration and (e) TiBr_4_ pulse duration. (f) AgBr film thickness as a function of deposition cycles. Unless otherwise evident the depositions in (d)–(f) were made with 800 cycles of 1.5 s long pulses and 1.0 s long purges at 110 °C. GPC of AgCl thin films as a function of (g) Ag­(fod)­(PEt_3_) pulse duration and (h) TiCl_4_ pulse duration. (i) AgCl film thickness as a function of deposition cycles. Unless otherwise evident the depositions in (g)–(i) were made with 800 cycles of 1.5 s long pulses and 1.0 s long purges at 105 °C. Note: The lines in the figures are meant for a guide to the eye.

Figure 4c,f,i show the film thicknesses as a function of the number of deposition cycles. For AgBr and AgCl (Figure 4f,i, the thickness increases almost linearly with the number of deposition cycles. However, AgI shows a clear deviation from the linearity (Figure 4c). The GPC for AgI is approximately 0.75 Å up to 800 cycles, and then decreases to around 0.55 Å. This change in the GPC occurs when the films become continuous (Figure 5b). The initial high GPC can be attributed to the roughness of the films or higher GPC on Si versus AgI. Films with a rough surface have a larger surface area than smoother films. Therefore, the initial film, consisting of islands, provides more adsorption and reaction sites for the precursors. As the film becomes continuous, the surface roughness and surface area decrease, leading to a decrease in the GPC.^36^ As shown in Figure 5a–c, the AgI film becomes continuous after approximately 800 cycles, at which point the film thickness is around 60 nm. The change in the GPC is observed around this cycle number (Figure 4c). In contrast, neither AgBr (Figure 5d–f) nor AgCl (Figure 5g–i) films reached continuity even after 800 cycles. This could explain why AgBr and AgCl exhibit constant GPC.

**Figure 5:**
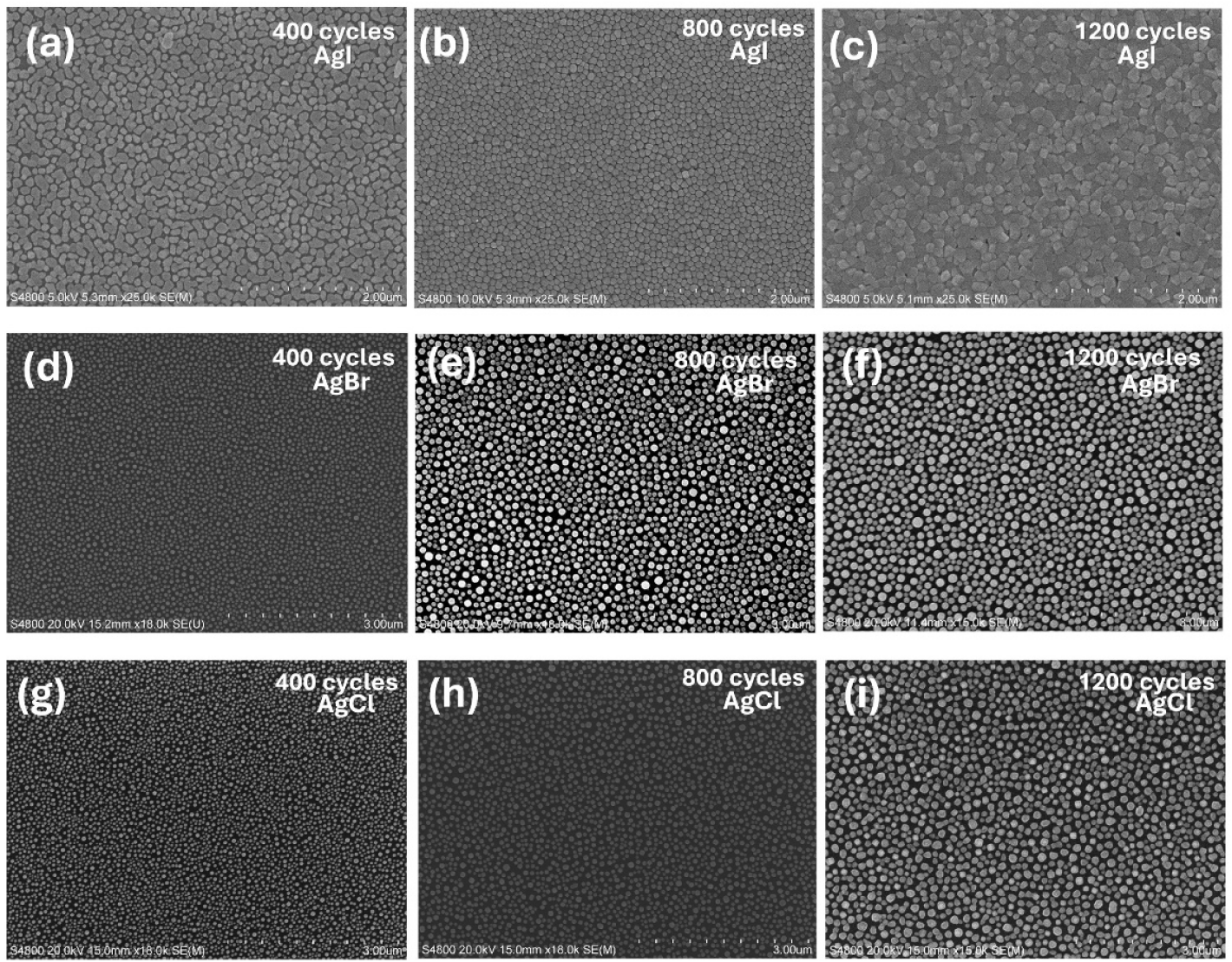
SEM images of (a–c) AgI (the depositions in (a), (b), and (c) were made at 105 °C with pulse times of 2.0, 1.5, and 2.0 s, and purge times of 2.0, 1.0, and 2.0 s, respectively), (d–f) AgBr (depositions in d–f were made with 1.5 s long pulses and 1.0 s long purges at 110 °C), and (g–i) AgCl (depositions in g–i were made with 1.5 s long pulses and 1.0 s long purges at 105 °C), at different cycle numbers.

The crystallinity of the silver halide films deposited at different temperatures using TiX_4_ precursors was studied. The GI-XRD patterns of the AgI films deposited from TiI_4_ (Figure 6a) exhibit sharp peaks corresponding to the hexagonal β-AgI phase (ICDD No. 09-0374). No significant changes in peak intensities or positions were observed as the deposition temperature was changed. Figure 6b shows the XRD patterns of the AgBr films deposited using TiBr_4_, with diffraction peaks corresponding to the cubic AgBr (ICDD No. 06-0438). The most intense reflection at 30.9° corresponds to the (200) plane. The XRD patterns of the AgBr films deposited at 105, 135, and 195 °C are similar, showing no significant changes. However, for the film deposited at 165 °C, the intensity of the (200) peak decreases notably and the (311) plane at 52.5° becomes relatively more intense. As discussed earlier, the XRD patterns of AgCl films deposited using TiCl_4_ display a single (111) reflection, indicating a highly preferential orientation (Figure 2). The AgCl films deposited using TiCl_4_ at various temperatures show very similar XRD patterns (Figure 6c).

**Figure 6:**
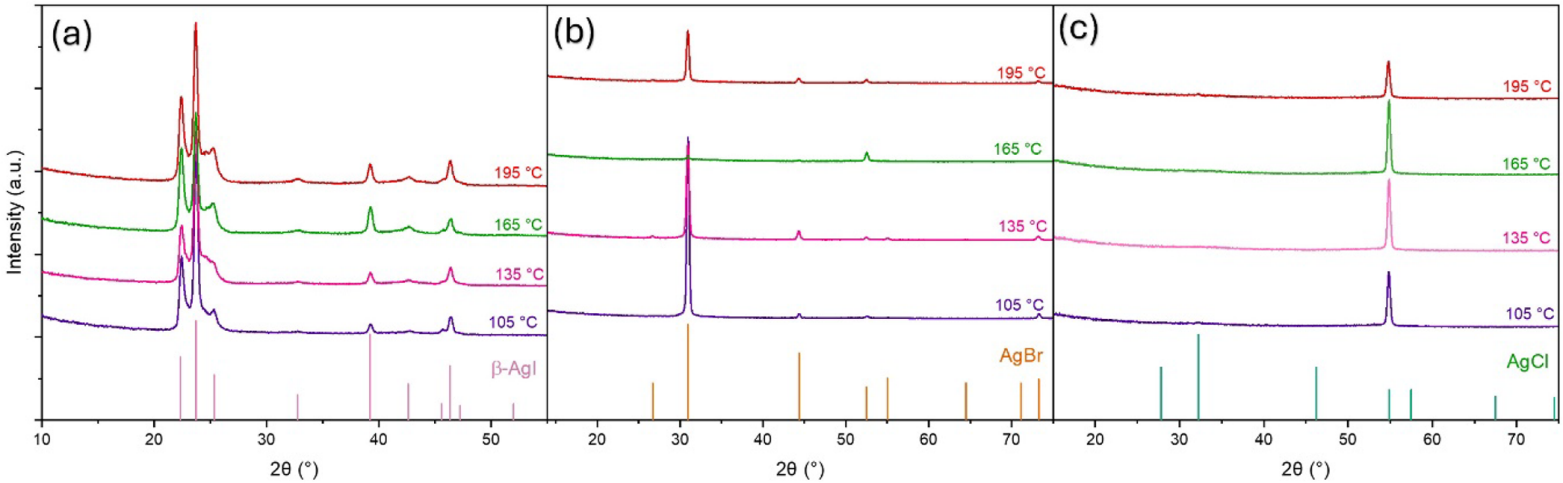
XRD patterns of (a) AgI, (b) AgBr, and (c) AgCl deposited using TiI_4_, TiBr_4_, and TiCl_4_ precursors at different temperatures.

Elemental analysis using ToF-ERDA was carried out to characterize the stoichiometry and impurity content of AgI, AgBr, and AgCl films resulting from the TiX_4_ precursors. The ToF-ERDA depth profiles are shown in Figure S10. All the films eroded during the ToF-ERDA measurements. The AgCl film exhibited a higher erosion rate compared to AgI and AgBr. The erosion of ALD halide films during ToF-ERDA has already been reported for metal fluoride films such as ScF_3_ and NiF_2_.^37,38^ The erosion was taken into account in the quantitative analysis. Table 2 summarizes the elemental compositions and X/Ag ratios of AgI, AgBr, and AgCl films deposited using TiX_4_ precursors determined by ToF-ERDA. The results confirm that the films are stoichiometric. The halogen-to-silver ratios were close to unity, which confirms stoichiometric compositions of all the films. ToF-ERDA also revealed the presence of oxygen and small amounts of other impurities. Hydrogen and oxygen likely originate from surface moisture.^39^ In addition, oxygen impurities may also originate from the metal–oxygen bonds in the beta-diketonate ligands of the silver precursors, as well as from postdeposition contamination due to air exposure. Remarkably, no titanium was detected in any of the films; a detection limit of 0.2 at.% is estimated for Ti in the AgX films.

**Table 2:** Elemental Composition of AgI, AgBr, and AgCl Films Deposited from TiX_4_ Precursors on Si Substrates as Measured with ToF-ERDA^a^

Films	Element atom %	X/Ag ratio
AgI	Ag 46.2%, I 47.5%, H 1.0%, C 0.9%, N 0.1%, O 4.3%	I/Ag = 1.03
AgBr	Ag 48.1%, Br 47.6%, H 0.8%, C 0.5%, O 3.0%	Br/Ag = 0.99
AgCl	Ag 43.6%, Cl 41.4%, H 0.5%, C 0.5%, O 9.0%, Br 5.0%	Cl/Ag = 0.95

a All films were deposited with 800 cycles. AgI deposition was done with 2.0 s long pulses and purges at 105 °C, AgBr done with 1.5 s pulses and 1.0 s purges at 110 °C, and AgCl deposition with 1.5 s pulses and 1.0 s purges at 105 °C.

To validate the potential application of ALD-deposited AgI film, we characterized its optical properties. As shown in Figure S11a, the UV–Vis transmittance spectrum shows an absorption edge at approximately 430–450 nm. The optical bandgap determined from the Tauc plot (Figure S11b) is about 2.8 eV, which is in good agreement with literature values for β-AgI (2.7–2.9 eV).^40,41^ These results illustrate that the deposited AgI films exhibit negligible absorption in the visible region up to the band edge and begin to absorb strongly near 2.8 eV in the UV region. This makes them promising candidates for optoelectronic applications that can also serve as high-quality starting layers for ternary silver halides and quaternary silver-based double perovskites with precise film thickness and uniformity.

## 3 Conclusion

This study presents new ALD processes for the deposition of silver halide thin films, utilizing Ag­(fod)­(PEt_3_) as the silver source and TiX_4_, SnX_4_, GaX_3_, and HX (X = Cl, Br, I) as halide precursors. GI-XRD and EDS analyses showed that AgI, AgBr, and AgCl films obtained using TiI_4_, TiBr_4_, and TiCl_4_ precursors had superior purity compared to those deposited using SnX_4_, GaX_3_, and HX precursors. The elemental composition of the films deposited from TiX_4_ precursors were investigated by ToF-ERDA which revealed halogen-to-silver ratios close to unity. No titanium was detected in the films. The ALD processes of AgX using Ag­(fod)­(PEt_3_) and TiI_4_, TiBr_4_, and TiCl_4_ were optimized at deposition temperatures of 105, 110, and 105 °C, respectively. The differences in film continuity, crystallinity, and impurities correspond with the surface adsorption and reactivity of the precursors. Although the exact atomic-scale reactions remain to be elucidated, these observations highlight the importance of precursor selection and deposition parameters. Among the halide precursors, TiI_4_ resulted in films with a consistent GPC, high crystallinity, and excellent continuity. The results highlight the capabilities of ALD for achieving precise control over the film growth and composition. This study provides a foundation for the development of ALD processes intended to support the applications of silver halides in next-generation technologies.

## 4 Experimental Section

### 4.1 Materials

The following precursors were used in this study: (2,2-dimethyl-6,6,7,7,8,8,8-heptafluorooctane-3,5-dionato)­silver­(I)-triethyl-phosphine (Ag­(fod)­(PEt_3_)) was synthesized in-house using literature methods.^42^ Titanium iodide (abcr, 99.9%), titanium bromide (Sigma-Aldrich, 98%), titanium chloride (Sigma-Aldrich, 98%), gallium iodide (abcr, 99%), gallium bromide (abcr, anhydrous), gallium­(III) chloride (abcr, 99.999%), tin iodide (Thermo Scientific Chemicals, 99+%, −10 mesh), tin bromide (Sigma-Aldrich, 99%), tin chloride (STREM, 98%), hydroiodic acid (Sigma-Aldrich, 57 wt % in H_2_O, 99.99%), hydrobromic acid (Sigma-Aldrich, 48 wt % in H_2_O, 99.99%), and hydrochloric acid (Sigma-Aldrich, ∼1.25 M in ethanol). All chemicals from commercial sources were used as received without further purification.

### 4.2 Film Deposition

The silver halide films were grown in a hot-wall flow-type F120 ALD reactor (ASM Microchemistry Ltd.) at a reactor pressure of approximately 5 mbar. Precursor pulsing and purging were done using inert gas valving. Nitrogen gas, purified using a Millipore Entegris Mykrolis WPMV200SI purifier (<1 ppb O_2_, < 1 ppb H_2_O at the outlet), was utilized as both the carrier and purging gas. Table 3 summarizes the delivery parameters for all the precursors used in the processes, including source temperature, container type, and delivery method.

**Table 3:** Parameters Used for Precursor Delivery^a^

Precursor	Source temperature	Source type	Note
Ag(fod)(PEt_3_)	95 °C	Open glass boat inside the reactor	
TiI_4_	105 °C	Open glass boat inside the reactor	
TiBr_4_	RT	Glass boat with an orificeinside the reactor	Rectangular orifice 5 × 5 mm
TiCl_4_	RT	Vapor draw from an external bottle/container	NV 0.5 turns
SnI_4_	75 °C	Open glass boat inside the reactor	
SnBr_4_	RT	Orifice glass boat inside the reactor	Rectangular orifice 5 × 5 mm
SnCl_4_	RT	Vapor draw from an external bottle/container	NV 1.25 turns
GaI_3_	65 °C	Open glass boat inside the reactor	
GaBr_3_	65 °C	Open glass boat inside the reactor	
GaCl_3_	RT	Orifice glass boat inside the reactor	Circular orifice (*d* = 1 mm) Dissolved in hexane in the glovebox, transferred to the boat via syringe, hexane evaporates during reactor pumpdown.^23^
HI(aq)	RT	Vapor draw from an external bottle/container	NV fully open
HBr(aq)	RT	Vapor draw from an external bottle/container	NV fully open
HCl(EtOH)	RT	Vapor draw from an external bottle/container	NV 1 turn

a Swagelok needle valve (NV) ss-4BMG was used.

Film depositions were carried out on native oxide covered silicon substrates (100 orientation, 5 × 5 cm^2^, Okmetic). The deposition process employed consecutive pulses of Ag­(fod)­(PEt_3_) and halide precursors, separated by nitrogen purging. The precursor pulse durations were between 1.0 and 4.0 s, while purge durations ranged from 1.0 to 2.0 s.

### 4.3 Film Characterization

A Hitachi S-4800 field-emission scanning electron microscope (FESEM) was used to evaluate surface morphologies of the deposited films. Energy-dispersive X-ray spectroscopy (EDS) measurements were conducted with an Oxford INCA 350 system coupled to the FESEM. Subsequently, the thicknesses of the films were determined from the EDS results using GMRFilm software and the bulk densities of AgI (5.68 g/cm^3^), AgBr (6.47 g/cm^3^), and AgCl (5.56 g/cm^3^). The thickness errors were calculated based on the uncertainty in the weight percentages from the EDS results and applying standard error propagation techniques. The elemental composition of the films was quantified with EDS using the emission lines of ILα, BrLα, ClKα, and AgLα.

The crystalline properties of the films were evaluated using grazing incidence X-ray diffraction (GI-XRD) on a Rigaku Smartlab diffractometer equipped with Cu Kα radiation (λ = 1.54 Å) and parallel beam optics. The incidence angle for GI-XRD was set to 1°.

Composition and depth profiles of the elements were studied by time-of-flight elastic recoil detection analysis (ToF-ERDA) using 35 MeV ^197^Au and 40 MeV ^127^I beams, with a measurement geometry of 16° + 24°. X-ray photoelectron spectroscopy (XPS) measurements were carried out using a PREVAC system with an Al Kα monochromatized anode (1486.7 eV) at a pressure of 10^–10^ mbar. Optical measurements (UV–vis) were conducted with a Hitachi U2000 spectrophotometer.

## Supplementary Material



## Notes

GI-XRD patterns of AgBr thin films deposited using SnBr_4_ at 135 and 165 °C (Figure S1); GI-XRD patterns of AgCl thin films deposited using GaCl_3_ at 135 and 165 °C (Figure S2); surface coverage ratios of silver halide films (Table S1); SEM images of AgBr films deposited from SnBr_4_ at different temperatures (Figure S3); SEM images of AgCl films deposited from GaCl_3_ at different temperatures (Figure S4); EDS spectra of AgI, AgBr, and AgCl films deposited from TiX_4_, GaX_3_, and SnX_4_ precursors (Figure S5); full XPS spectra of AgI, AgBr, and AgCl films deposited from TiX_4_, GaX_3_, SnX, and HX precursors (Figures S6–S8); growth per cycle (GPC) of AgI, AgBr, and AgCl thin films as a function of purge durations (Figure S9); ToF-ERDA elemental depth profiles (Figure S10); UV–Vis transmittance spectrum and Tauc plot of ALD β-AgI thin film (Figure S11) (PDF)
