# Supplementary material for: Atomic Layer Deposition of AgX (X = Cl, Br, I) Thin Films
Source: ACS Omega. 2025 Nov 14;10(46):55682–91. doi: 10.1021/acsomega.5c06913 (PMC12658672; doi:10.1021/acsomega.5c06913)
Supplement: Supplementary file 1 [file ao5c06913_si_001.pdf]

Supporting Information

Atomic Layer Deposition of AgX (X = Cl, Br, I) Thin Films

Aida Heidari[1]*, Georgi Popov[1], Timo Hatanpää[1], Alexander Weiß[1], Mykhailo Chundak[1], Kenichiro Mizohata[2], Mikko Ritala[1], Marianna Kemell[1]**

[1] Department of Chemistry, University of Helsinki, Helsinki, FI-00014 Finland

[2] Department of Physics, University of Helsinki, Helsinki FI-00014, Finland

Corresponding authors:

*Aida Heidari (aida.heidari@helsinki.fi)

**Marianna Kemell (marianna.kemell@helsinki.fi)

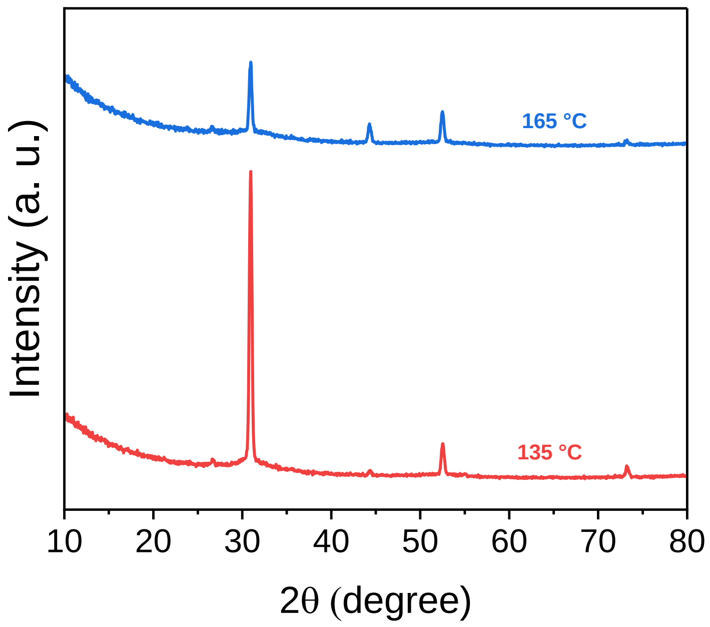

Figure S1. GI-XRD patterns of AgBr thin films deposited using $SnBr_4$ on Si substrate. The films were deposited at 135 and 165 °C with 800 cycles. Ag(fod)(PEt_3) pulse, $SnBr_4$ pulse, and purge durations were 1.5, 1.5, and 1.0 s respectively.

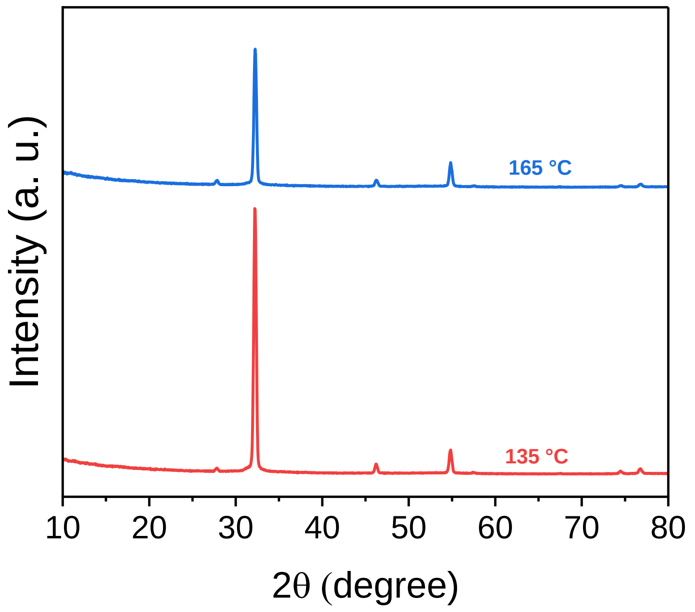

Figure S2. GI-XRD patterns of AgCl thin films deposited using $GaCl_3$ on Si substrate. The films were deposited at 135 and 165 °C with 800 cycles. Ag(fod)(PEt_3) pulse, $GaCl_3$ pulse, and purge durations were 1.5, 1.5, and 1.0 s respectively.

Table S1. Surface coverage ratios of silver halide films deposited at 105 °C with 800 ALD cycles using different halide precursors. Ratios were determined from SEM images using ImageJ software.

**Film**	**Precursor**	**Surface Coverage (%)**
AgI	TiI_4	97
	GaI_3	78
	SnI_4	91
	HI	78
AgBr	$TiBr_4$	54
	$GaBr_3$	99
	$SnBr_4$	79
	HBr	61
AgCl	$TiCl_4$	56
	$GaCl_3$	98
	$SnCl_4$	64
	HCl	51

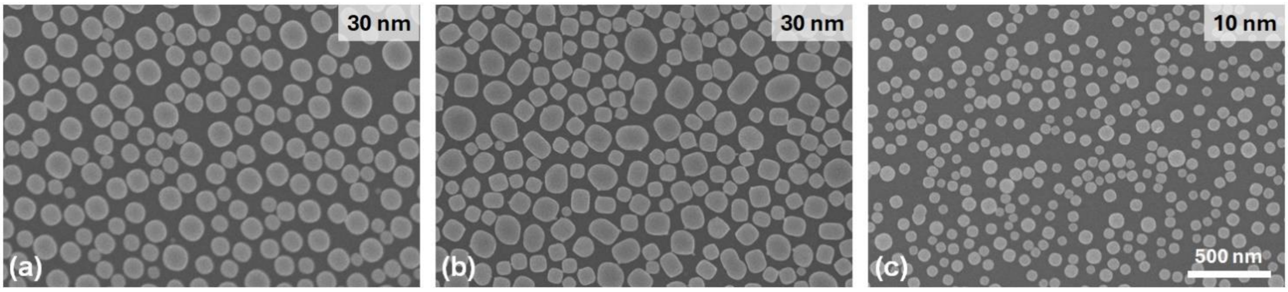

Figure S3. SEM images of AgBr films deposited from $SnBr_4$ at different temperatures: (a) 135 °C, (b) 165 °C, and (c) 195 °C. All films were deposited on Si with 800 cycles. The Ag(fod)(PEt_3) pulse, $SnBr_4$ pulse, and purge durations were 1.5, 1.5, and 1.0 s, respectively.

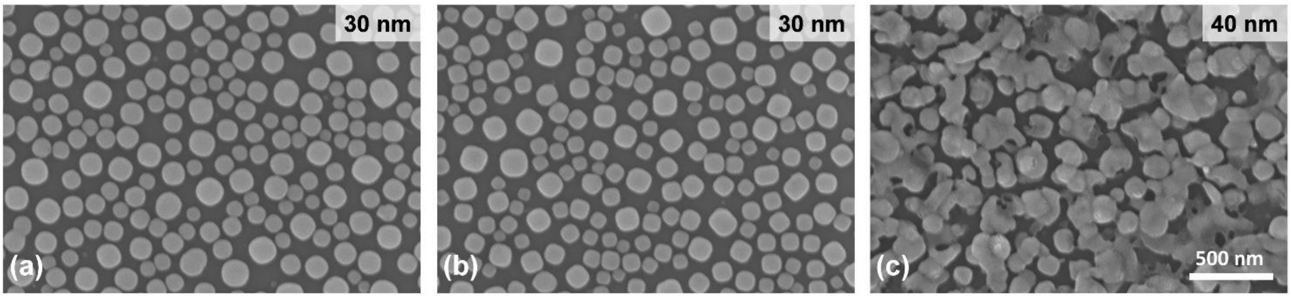

Figure S4. SEM images of AgCl films deposited from $GaCl_3$ at different temperatures: (a) 135 °C, (b) 165 °C, and (c) 195 °C. All films were deposited on Si with 800 cycles. The Ag(fod)(PEt_3) pulse, $GaCl_3$ pulse, and purge durations were 1.5, 1.5, and 1.0 s, respectively.

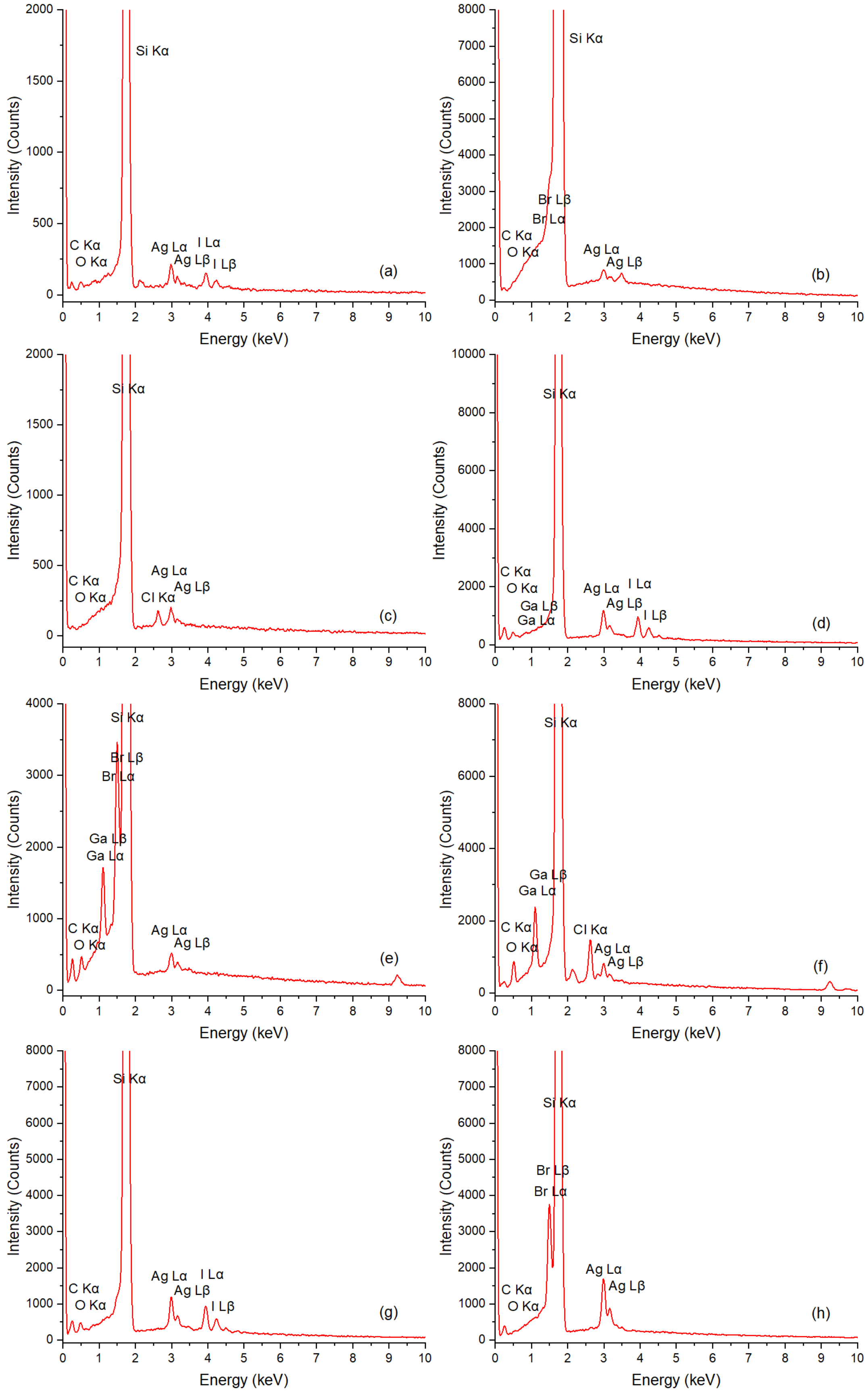

Figure S5. EDS spectra of AgI, AgBr, and AgCl films deposited from TiI_4 (a), $TiBr_4$ (b), and $TiCl_4$ (c), respectively; EDS spectra of AgI, AgBr, and AgCl films deposited from GaI_3 (d), $GaBr_3$ (e), and $GaCl_3$ (f), respectively; EDS spectra of AgI and AgBr films deposited from SnI_4 (g) and $SnBr_4$ (h), respectively.

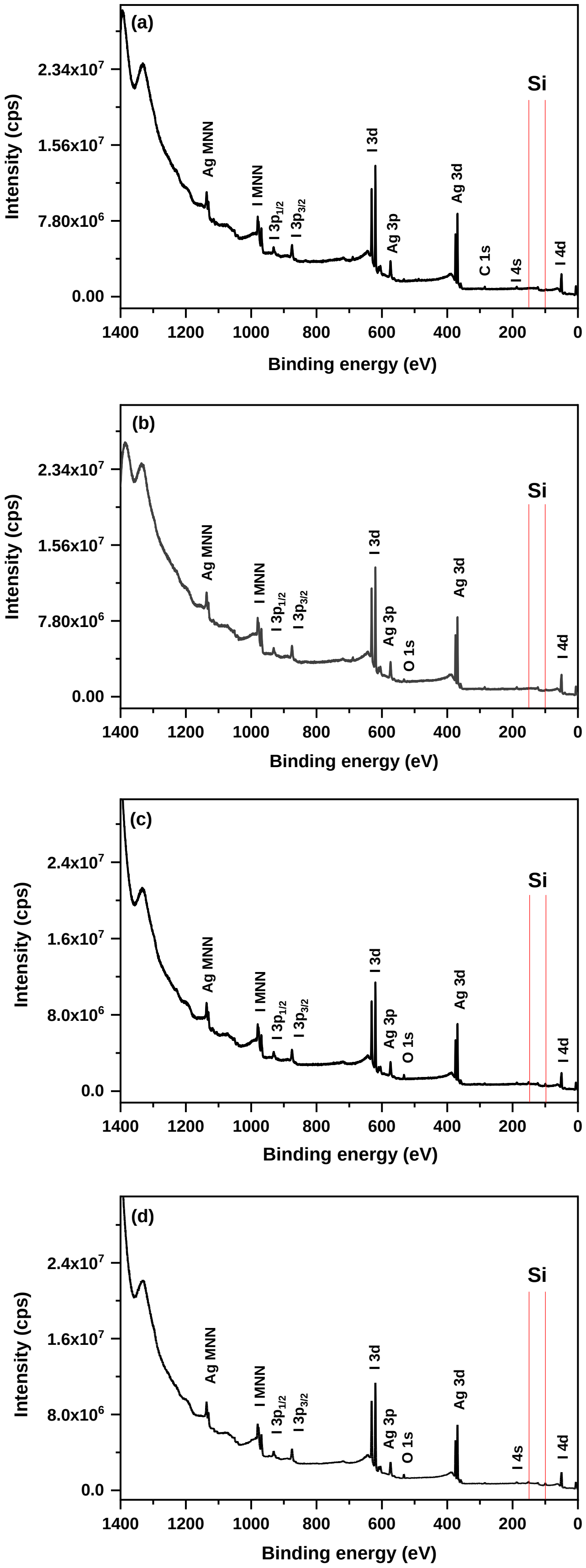

Figure S6. Full XPS spectra of AgI deposited from TiI_4 (a), GaI_3 (b), SnI_4 (c), and HI (d) precursors. Depositions were done with 800 cycles, pulse durations of 1.5 s for both Ag(fod)(PEt_3) and halide precursors, and purge durations of 1.0 s.

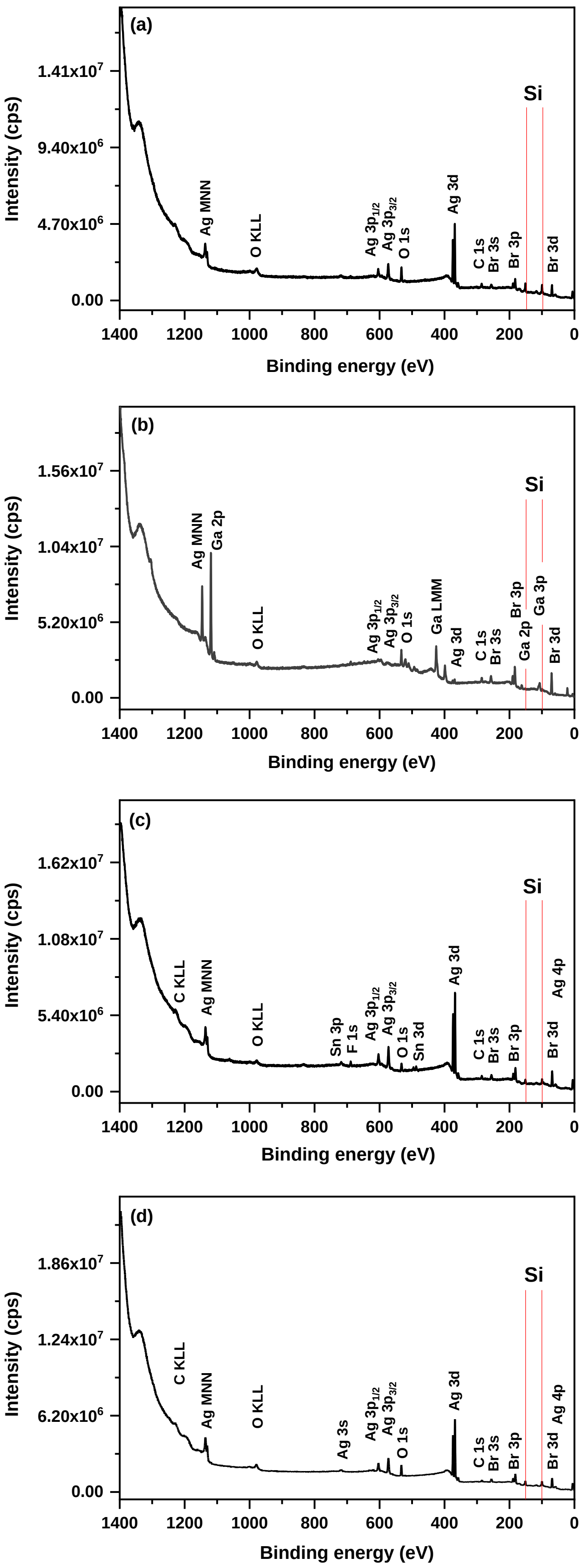

Figure S7. Full XPS spectra of AgBr deposited from $TiBr_4$ (a), $GaBr_3$ (b), $SnBr_4$ (c), and HBr (d) precursors. Depositions were done with 800 cycles, pulse durations of 1.5 s for both Ag(fod)(PEt_3) and halide precursors, and purge durations of 1.0 s.

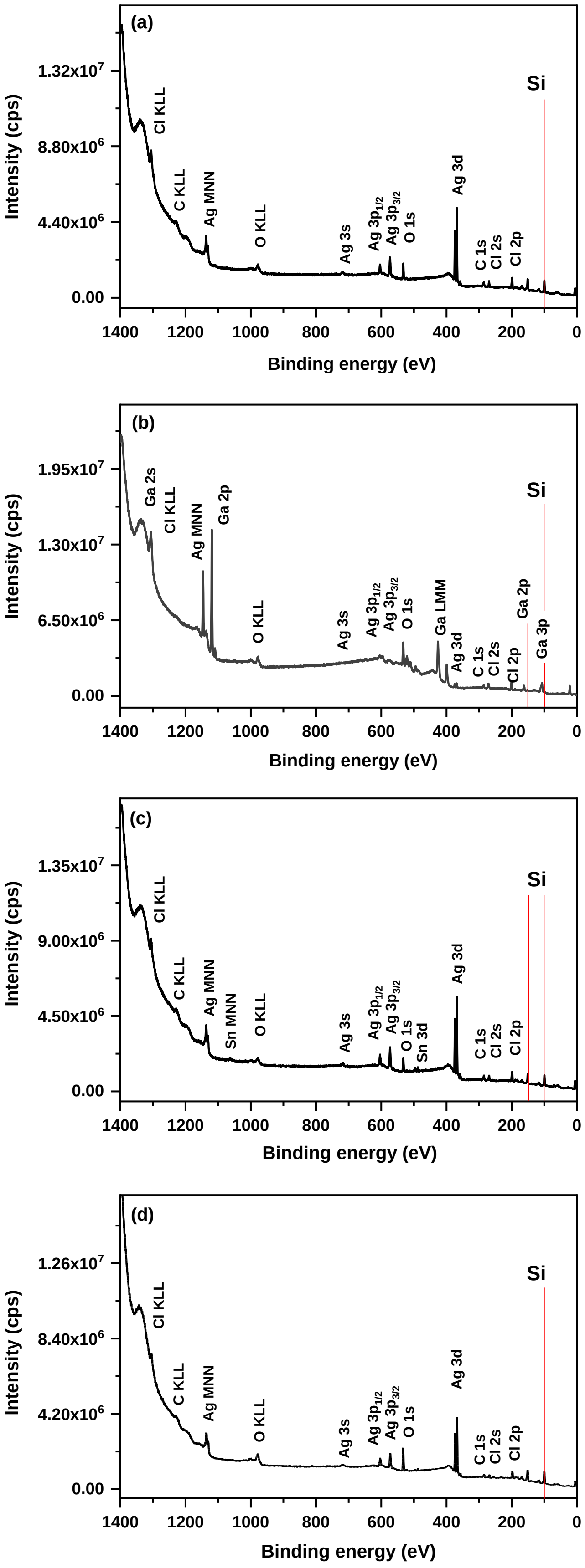

Figure S8. Full XPS spectra of AgCl deposited from $TiCl_4$ (a), $GaCl_3$ (b), $SnCl_4$ (c), and HCl (d) precursors. Depositions were done with 800 cycles, pulse durations of 1.5 s for both Ag(fod)(PEt_3) and halide precursors, and purge durations of 1.0 s.

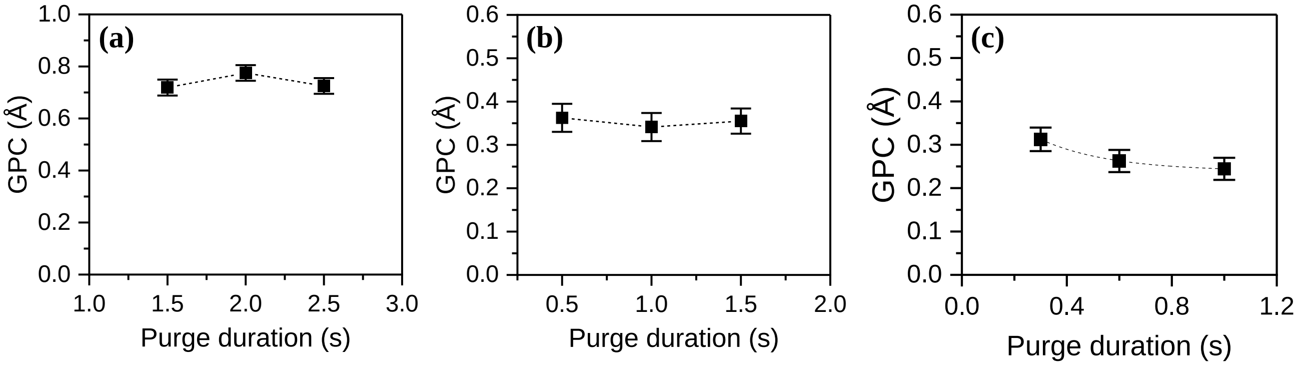

Figure S9. GPC of (a) AgI, (b) AgBr, and (c) AgCl thin films as a function of purge durations. The depositions in (a) were made with 800 cycles of 2.0 s long pulses at 105 °C; the depositions in (b) were made with 800 cycles of 1.5 s long pulses at 110 °C; the depositions in (c) were made with 800 cycles of 1.5 s long pulses at 105 °C.

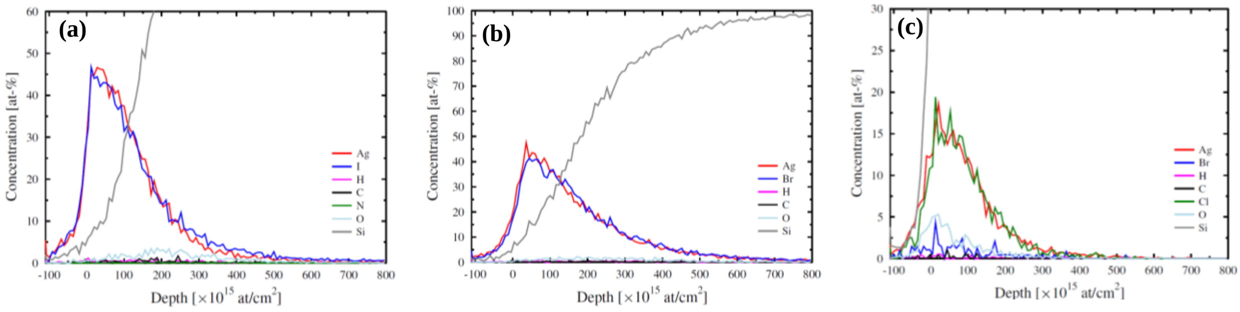

Figure S10. ToF-ERDA elemental depth profiles of (a) AgI, (b) AgBr, and (c) AgCl deposited using TiI_4, $TiBr_4$, and $TiCl_4$ precursors at 105 °C.

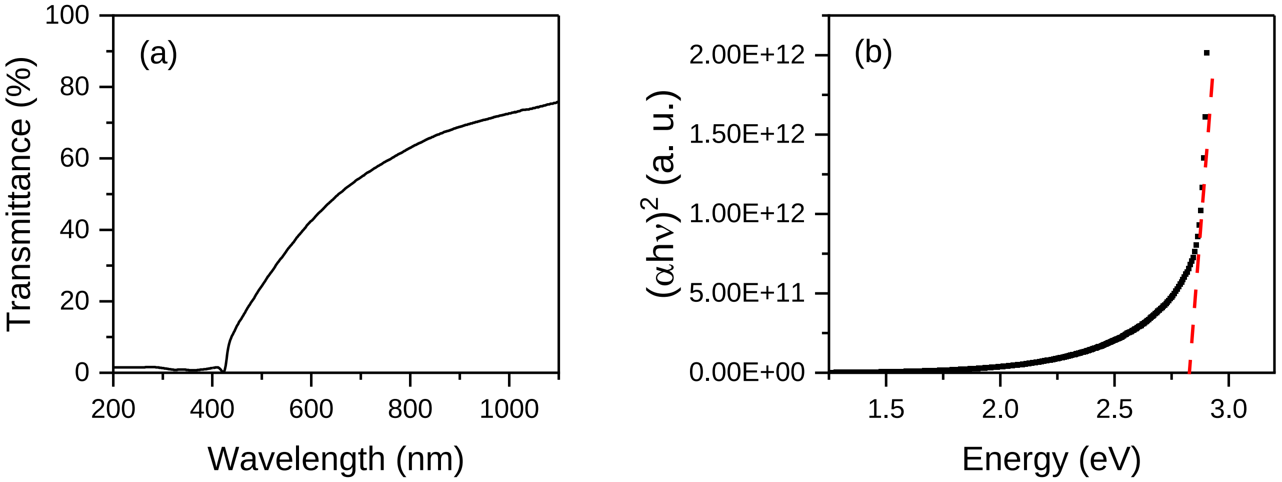

Figure S11. (a) UV–Vis transmittance spectrum and (b) Tauc plot of ALD β-AgI thin film.
